# Leg ulcer in a patient with Rothmund–Thomson syndrome

**DOI:** 10.1186/s40064-015-1374-z

**Published:** 2015-10-05

**Authors:** Sinem Ciloglu, Alpay Duran, Sirin Yasar Pekcan, Hasan Buyukdogan

**Affiliations:** Department of Plastic, Reconstructive and Aesthetic Surgery, Haydarpasa Numune Training and Research Hospital, Istanbul, Turkey; Department of Plastic, Reconstructive and Aesthetic Surgery, Sinop Ataturk State Hospital, Sinop, Turkey; Department of Dermatology, Haydarpasa Numune Training and Research Hospital, Istanbul, Turkey

## Abstract

**Background:**

Rothmund–Thomson syndrome is a rare genetic condition exhibiting some dermatological, craniofacial, ophthalmological, and central nervous system abnormalities.

**Case description:**

A 51-year-old male patient, diagnosed with Rothmund–Thomson syndrome, attended to our outpatient clinic with complaint of unhealing wound in lower part of his left leg. Over this period, he had received various local therapies such as creams, wound dressings and hyperbaric oxygen therapy but no progress could be achieved. The wound gradually enlarged. Negative pressure wound therapy was applied at −125 mmHg for 20 days. Wound was finally covered with split-thickness skin graft.

**Discussion and evaluation:**

There is only one case of Rothmund–Thomson syndrome with leg ulcer reported in the literature. However, complete closure has not been achieved with non-surgical therapies in this case. Therefore we performed negative pressure wound therapy followed by skin grafting.

**Conclusions:**

It is useful to treat therapy resistant wounds in Rothmund–Thomson syndrome by negative pressure, which can preserve residual vital tissue, and help clear away necrotizing tissue effectively and close the wound promptly.

## Background

Rothmund–Thomson syndrome (RTS) is an autosomal recessive condition presenting in infancy with specific facial rashes and heterogeneous clinical characteristics including short stature, hair and nail abnormalities, photosensitivity, sparse or absent eyelashes and/or eyebrows, juvenile cataracts, skeletal changes, predisposition to osteosarcoma and skin cancer (Larizza et al. 2010). RTS has been categorized into genetic cancer predisposition disorders that fall into the class of DNA repair or chromosomal instability disorders. Here, we report a case of a therapy-resistant leg ulcer in a patient with RTS successfully treated by negative pressure wound therapy (NPWT) and skin grafting.

## Case description

**Fig. 1:**
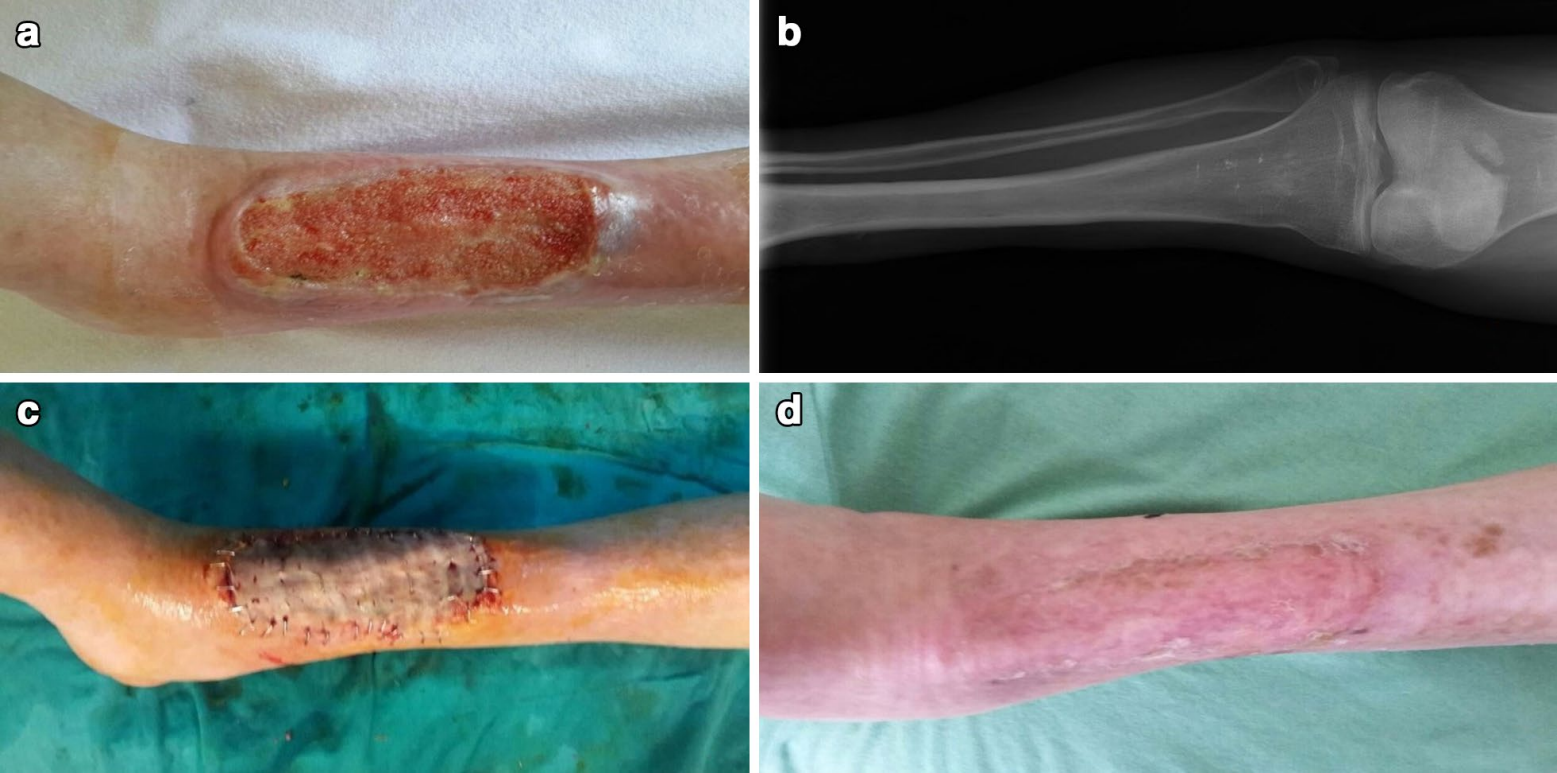
**a** Preoperative view of the case. **b** Preoperative X-ray of the left leg with osteopenia. **c** Early postoperative view of the case. **d** Late postoperative view of the case after 6 month

A 51-year-old male patient, diagnosed with RTS, was referred to our clinic with un-healing wound in lower part of his left leg. His wound had occurred following a trauma and had been present for over 6 months. Over this period, he had received various local therapies such as topical medications, wound dressings and hyperbaric oxygen therapy but no progress could be achieved. Inevitably, the wound gradually enlarged (Fig. 1a). His physical examination showed saddle nose, maxillary hypoplasia, small hands and feet. The muscles of both upper and lower extremities were atrophic, especially on the distal regions. X-ray studies showed osteopenia in every bony structure (Fig. 1b). Femoral, popliteal and pedal pulses were palpable in both lower extremities. An elliptical shaped, sharp-bordered, 20 cm × 10 cm sized wound was noted on the distal anterior aspect of patient’s left leg. An incisional biopsy was performed but no neoplastic changes were observed. Microbiological studies of the wound were performed during the treatment and follow-up periods, but no pathogen microorganism was cultured.

After preoperative assessment and proper wound debridement, extensor tendons were exposed. NPWT was applied at −125 mmHg for 20 days and granulation tissue formed over tendons. Wound was finally covered with split-thickness skin graft harvested from left thigh and the graft healed completely (Fig. 1c, d).

## Discussion and evaluation

In humans, mutations in three of the five members of the RecQ family lead to separate genetic diseases as Bloom, Werner, and RTS. RTS is caused by homozygous or compound heterozygous mutations in RECQL4 DNA helicase gene mapped on chromosome 8q24.3 (Larizza et al. 2010). Some cytogenetic studies from patients with RTS showed clonal/non-clonal structural abnormalities of chromosome 8 and presence of DNA repair defect in lymphocytes and fibroblasts. Chromosomal or genomic instability and abnormal DNA repair have been held responsible for both premature aging and increased risk of skin and skeletal cancers in these patients (Wang et al. 2001).

A chronic leg ulcer in a patient with RTS is alarming for an early stage skin cancer. Skin biopsies are important for determining the etiology and excluding occasional cases of malignancies presenting as leg ulcers. Fortunately, there is no evidence of neoplastic change in our case. Chronic leg ulcer in RTS is related to reduced DNA repair capacity, chromosomal instability of fibroblasts and lymphoblasts and reduced restoration of skin cell structures, particularly faulty collagen (Altunay et al. 2010).

There is only one case of RTS with leg ulcer in the English literature. Complete covering could not been achieved with non-surgical therapies in this case (Altunay et al. 2010). We preferred to apply NPWT followed by skin grafting for our case. NPWT stimulates wound healing through optimization of blood flow, decreasing tissue edema and removing excessive fluid. These changes facilitate the removal of bacteria from the wound bed. Furthermore, the application of sub-atmospheric pressure alters the cytoskeleton of the cells in the wound bed through micro-strain, triggering a cascade of intracellular signals that increase the rate of cell division and subsequent formation of granulation tissue (Seidel et al. 2014). NPWT has been used in over 861 peer-reviewed journals across all medical and surgical specialties, demonstrating its potential in acute and chronic wounds and post-operative recovery on January 2014 (KCI website Accessed: 25th February 2015). Applying NPWT to a newly laid down skin graft is common practice, with a number of studies showing an improvement in graft incorporation using a pressure range between −50 and −80 mmHg. Loss of partial-thickness skin graft has been shown to be consistently lower when compared to standard bolstering (Webb 2002).

## Conclusions

RTS is a rare syndrome and there has been a limited number of RTS cases with leg ulcer reported in literature. In this study, we achieved to close the wound of a therapy-resistant leg ulcer patient with RTS using NSWT and STSG.

## Consent

Written informed consent was obtained from the patient for the publication of this report and any accompanying images.

## Abbreviations

RT: Rothmund–Thomson syndrome
NPWT: negative pressure wound therapy
